# Myopia Outcome Study of Atropine in Children (MOSAIC): an investigator-led, double-masked, placebo-controlled, randomised clinical trial protocol

**DOI:** 10.12688/hrbopenres.12914.1

**Published:** 2019-07-23

**Authors:** Saoirse McCrann, Ian Flitcroft, Niall C. Strang, Kathryn J. Saunders, Nicola S. Logan, Samantha Szeyee Lee, David A. Mackey, John S. Butler, James Loughman

**Affiliations:** 1 Centre for Eye Research Ireland, School of Physics, Clinical and Optometric Sciences, Technological University Dublin, Ireland, Dublin, Ireland; 2 Children’s University Hospital, Dublin, Ireland; 3 Department of Vision Sciences, Glasgow Caledonian University, Glasgow, UK; 4 School of Biomedical Sciences, University of Ulster, Coleraine, UK; 5 Optometry & Vision Science Research Group, Aston Optometry School, Aston University, Birmingham, UK; 6 Centre for Ophthalmology and Visual Science (incorporating Lions Eye Institute), University of Western Australia, Perth, WA, Australia; 7 School of Mathematical Sciences, Technological University Dublin, Dublin, Ireland

**Keywords:** myopia, atropine, myopia progression, myopia control

## Abstract

**Background: **The Myopia Outcome Study of Atropine in Children (MOSAIC) aims to explore the efficacy, safety, acceptability and mechanisms of action of 0.01% unpreserved atropine for myopia control in a European population.

**Methods: **MOSAIC is an investigator-led, double-masked, placebo-controlled, randomised clinical trial (RCT) investigating the efficacy, safety and mechanisms of action of 0.01% atropine for managing progression of myopia. During Phase 1 of the trial, 250 children aged 6-16 years with progressive myopia instil eye drops once nightly in both eyes from randomisation to month 24. No treatment is given during Phase 2 from month 24 to 36 (washout period) for those participants initially randomised to the intervention arm (n=167), during which any potential rebound effects on cessation of treatment will be monitored. All participants initially assigned to the placebo (n=83) crossover to the intervention arm of the study for Phase 2, and from month 24 to 36, instil 0.01% atropine eye drops in both eyes once nightly. Further treatment and monitoring beyond 36 months is planned (Phase 3) and will be designed dependent on the outcomes of Phase 1.

**Results: **The primary outcome measure is cycloplegic spherical equivalent refractive error progression at 24 months. Secondary outcome measures include axial length change as well as the rebound, safety and acceptability profile of 0.01% atropine. Additional analyses will include the mechanisms of action of 0.01% atropine for myopia control.

**Conclusions: **The generalisability of results from previous clinical trials investigating atropine for myopia control is limited by the predominantly Asian ethnicity of previous study populations. MOSAIC is the first RCT to explore the efficacy, safety and mechanisms of action of unpreserved 0.01% atropine in a predominantly White population.

**Trial registration: **ISRCTN: ISRCTN36732601 (04/10/2017), EudraCTdatabase 2016-003340-37 (03/07/2018).

## Introduction

Myopia is expected to affect 2.5 billion people by 2020 and close to 5 billion by 2050 ^1, 2^. Developed countries in East and Southeast Asia have the highest prevalence of adolescent myopia, with over 90% of 18 year olds in Singapore and 72% in China affected ^3^. There is also evidence that myopia prevalence is increasing in Europe, with myopia affecting almost 50% of 25 to 29 year olds, and the proportion of myopic children more than doubling in the UK over the last 50 years ^4– 6^. Likewise, in the past 30 years, myopia prevalence has almost doubled in the United States affecting almost 50% of school-leavers ^7^. Standard clinical care of myopia progression, however, only addresses the optical impact of this condition, rather than treating its underlying biological basis ^8^.

Epidemiological studies indicate that myopia is second only to age as a risk factor for several eye diseases including glaucoma, cataract and retinal detachment ^9^, and is the primary risk factor in myopic maculopathy ^10^. Myopic maculopathy is a leading cause of blindness throughout Asia ^11^ and has been consistently shown as a major cause of blindness among the working age population across Europe ^12– 16^. Myopic maculopathy is a leading cause of blindness, there is no available treatment, no proven aetiological basis and relatively little research funding.

Research in several Asian countries including Singapore, China, Korea and Taiwan has demonstrated that atropine eye drops significantly slow myopia progression ^17– 22^. A recent network meta-analysis suggests that atropine, at various doses, is the most effective therapy for controlling refractive error and axial eye growth progression ^23^. As the risk of complications increases monotonically with the degree of myopia ^9^, slower progression has the potential to substantially reduce the prevalence of myopia-related vision impairment and the associated quality-of-life and socio-economic impacts.

Administered as an eye drop, atropine blocks the muscarinic receptors in the pupillary sphincter musculature, causing pupillary dilatation (mydriasis) which can induce symptoms of photophobia. Atropine also reduces or paralyses contraction of the ciliary muscle resulting in blurred proximal vision due to loss of accommodation (cycloplegia) at its normal 0.5% or 1% clinical dose. The severity and persistence of these atropine effects are dose-dependent ^18^, hence lower doses would be expected to have lesser mydriatic and cycloplegic effect. The principal justification for using 0.01% atropine for myopia control comes as a serendipitous finding from the Atropine Treatment Of Myopia 2 (ATOM2) study ^18^. Some of the key findings of ATOM2 included that 0.01% atropine achieved excellent control of refractive error progression during the initial treatment phase ^18^, and ultimately demonstrated the best balance of clinical efficacy and side-effect (cycloplegia and mydriasis related) profile over the entire five-year study duration ^24^. 

There are a number of unresolved issues, however, regarding the use of atropine as a myopia control measure. Firstly, most of the atropine studies have been conducted in Asia, thus the results of which cannot be simply extrapolated to other populations, particularly given that atropine exhibits a high affinity for melanin ^25^. Although 0.01% atropine appears to be well tolerated in a Caucasian population exhibiting light irides ^26^, the longer-term safety, acceptability and efficacy of atropine in a Caucasian cohort has yet to be defined, indicating the need for suitably designed clinical trials of low-dose atropine including other ethnicities. Secondly, the ATOM2 study was not placebo-controlled, while the ongoing Low-Concentration Atropine for Myopia Progression (LAMP) study was only placebo-controlled for the first year ^27^.

The suggestion that 0.01% atropine is effective in slowing refractive changes in myopia progression has led many hospital ophthalmology departments, particularly in Asia, to switch from using higher doses to using 0.01% atropine. There is also increasing uptake of low dose atropine in private ophthalmology and optometry practice in many parts of the world ^28^. However, an apparent discordance in the two-year ATOM2 treatment outcomes is a concern. While 0.01% atropine apparently slowed refractive error progression, the rate of axial elongation in this treatment arm during the initial two-year treatment phase was marginally faster than that observed in the historical controls of the original ATOM1 placebo control group ^29^. The year one findings from the LAMP study also suggest a dose response effect in relation to the impact of atropine on axial growth, but the lack of placebo control in future years of this study eliminates the possibility of exploring this issue over a longer treatment time-course ^27^. The inconsistency between refractive and axial growth outcomes is an issue that can only be addressed by a placebo-controlled trial of sufficient duration to definitively address the use of 0.01% atropine for myopia control. Other unresolved questions include the site and mode of action, the optimal concentration and long-term safety of low dose atropine. Most of the available research has primarily focussed on efficacy of the drug ^30^. Therefore investigations to assess the effect of atropine on biometric characteristics such as choroidal thickness ^31^, retinal thickness ^31^, crystalline lens thickness ^32, 33^, anterior chamber depth ^32, 33^, optic disc parameters ^34^, corneal curvature ^33^, and axial length ^23^, should also be prioritised in order to advance our knowledge in relation to the mechanisms involved in the regulation of axial elongation.

Past clinical trials investigating atropine as a myopia control intervention have used preserved atropine formulations ^17, 18, 23^. Although it is possible that the preservative component of the drop may enhance penetration of the drug into the anterior chamber ^35, 36^, chronic application of eye drops containing preservatives can induce significant cytological and histological impairment in ocular tissues ^37– 39^, potentially leading to toxic adverse effects and non-tolerance to the eye drop ^40^. Such potential for chronic damage is not ideal for a drug that may be required for use over an extended period of time in a paediatric population. Evaluation of the safety and efficacy of an unpreserved formulation is an important feature of this trial.

The Myopia Outcome Study of Atropine In Children (MOSAIC) has been designed to address some of these important research questions. The overarching goal of the MOSAIC trial is to explore the efficacy, safety, acceptability and mechanisms of action of 0.01% atropine for myopia control in European children. Trial funding was awarded through the Medical Research Charities Group (MRCG) and funded by the Health Research Board (HRB) and Fighting Blindness, a non-profit organisation, under the MRCG-HRB Joint Funding Scheme [Grant Number: MRCG 2016-13]. The design and methodology of the MOSAIC trial is outlined herein.

## Methods

### Management, design and registration

**Figure 1.**
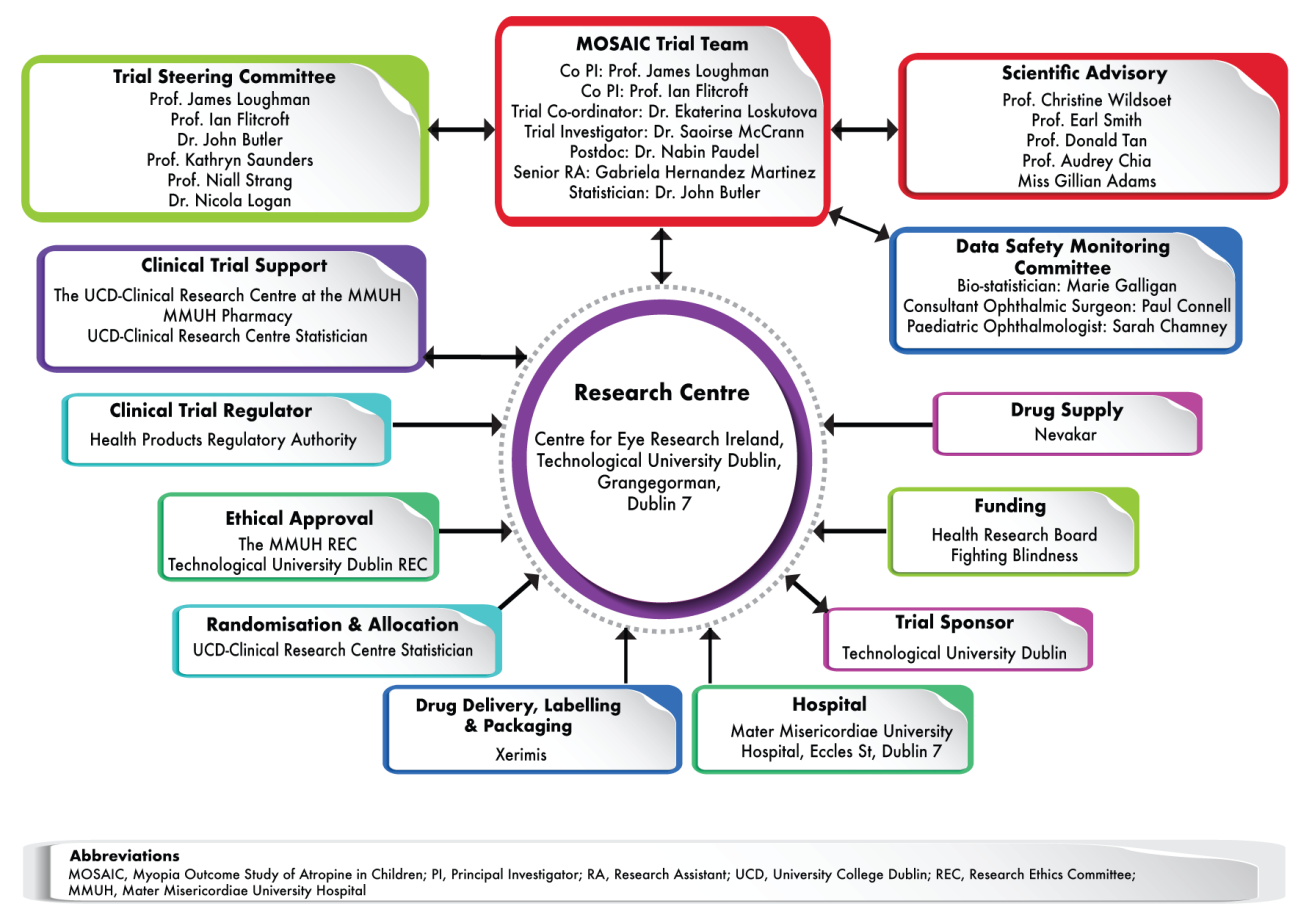
MOSAIC support and oversight structures.

The management team and relevant structures established to oversee the implementation of MOSAIC, including the data safety monitoring committee (DSMC), trial steering committee (TSC) and scientific advisory committee (SAC), are outlined in Figure 1.

**Figure 2.**
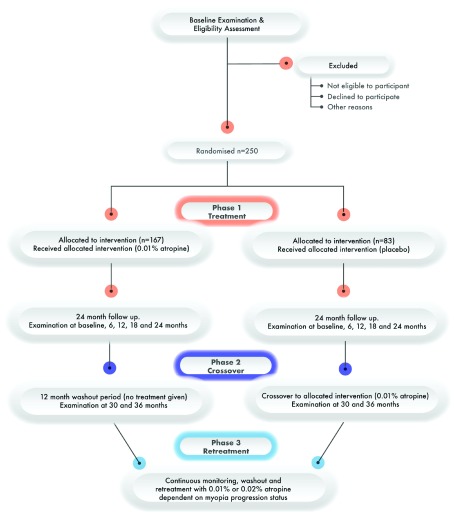
Flow of participants through the MOSAIC clinical trial.

MOSAIC is an investigator-led, double-masked, placebo-controlled, randomised clinical trial (RCT) designed to assess the efficacy, safety, acceptability and mechanisms of action of 0.01% atropine for controlling myopia in children. The trial protocol, developed according to Standard Protocol Items Recommendations for Interventional Trials (SPIRIT) guidelines, is registered on the current controlled trials register with the International Standard Randomised Controlled Trials Number 36732601 ( ISRCTN36732601) on 4 October 2017 and also on the EudraCTdatabase ( 2016-003340-37) on the 3 July 2018. The trial is a single centre study conducted at the Centre for Eye Research Ireland (CERI) at Technological University Dublin, Ireland. The progress of participants through the phases of MOSAIC are shown in Figure 2 in the consolidated standards of reporting trials (CONSORT) diagram ^41^. SPIRIT and CONSORT reporting checklists for MOSAIC are deposited in TU Dublin’s ARROW repository (see Reporting guidelines ^42, 43^).

### Patient and public involvement

The MOSAIC protocol and clinical trial documentation were designed to maximise the benefits of patient and public involvement (PPI) throughout the clinical trial ^44^. All MOSAIC clinical trial documentation, including consent/assent forms, participant information leaflets and parental questionnaires, were submitted through Fighting Blindness for PPI review. The collated feedback was analysed, and the relevant clinical trial documentation was amended to reflect PPI recommendations.

### Research question

To address whether it is clinically justifiable and feasible to offer atropine eye drops to limit the progression of myopia in a European population. This will be achieved by determining the efficacy, safety and acceptability of 0.01% atropine treatment.

### Primary outcome measure

Change in spherical equivalent refraction at 24 months measured by cycloplegic auto-refraction.

### Secondary outcome measures

1. Efficacy

• Change in ocular axial length at 24 months measured by optical low-coherence interferometry.
• Change in spherical equivalent refraction at 12 months measured by cycloplegic auto-refraction.
• Change in ocular axial length at 12 months measured by optical low-coherence interferometry.
• Percentage of participants who progress <0.25D (dioptre), 0.25D≤0.75D and >0.75D in 24 months.
• Rebound acceleration in myopic refractive error after cessation of atropine treatment, measured as change in spherical equivalent refraction and axial length between 24 and 36 months.
2. Mechanisms of action
• Effects on off-axis refraction measured by cycloplegic auto-refraction at 24 months.
• Effects on ocular growth (including retinal vascular morphology, ocular biometry, corneal topography, anterior chamber, lens thickness, retinal nerve fibre layer and choroidal thickness) at 24 months.
3. Safety and acceptability
• Changes in visual performance (distance and near visual acuity (VA), stereoacuity) at 24 months.
• Effects on ocular physiological response (amplitude of accommodation, accommodative facility, lag of accommodation, near point of convergence, pupil size and pupil reactivity) at 24 months.
• Quality of life impact associated with atropine use at 24 months.
• Frequency of adverse events recorded on study-specific report forms.
• Participant dropout rate during the trial.
• Compliance with trial medication.

### Drug manufacture and supply

Unpreserved atropine 0.01% is supplied by the pharmaceutical company Nevakar (Nevakar, Inc. 1019 US Highway 202-206, Building K, NJ Center of Excellence, Bridgewater, NJ 08807, USA) for the duration of the clinical trial. Nevakar’s contracted manufacturer, Excelvision, has a current EU GMPD Certificate and is authorised to manufacture atropine 0.01% and placebo eye drops on behalf of Nevakar, in accordance with the requirements of the EU Guide to Good Manufacturing Practice. The eye drops are manufactured at a site authorised for manufacture of aseptically prepared small volume liquids. Investigational product labelling, packaging and QP release to the trial centre is provided by Xerimis.

### Trial intervention

The intervention for the trial is 0.01% w/v atropine eyedrops, an anti-cholinergic agent selective for muscarinic receptors. Unpreserved 0.01% w/v atropine solution is supplied to trial participants as single dose ampoules for daily use. The placebo solution, identical to the atropine eye drop formulation without the active ingredient (atropine), is also supplied to trial participants as single dose ampoules for daily use.

During Phase 1 of MOSAIC, participants instil eye drops (atropine or placebo, per their randomisation status) once nightly into both eyes from the baseline visit to month 24. This is followed by Phase 2 where no treatment is given from month 24-to-36 (washout period) for those participants initially randomised to atropine. Participants are monitored in this washout period to examine any potential rebound effects on termination of treatment. All participants initially assigned to the placebo group then crossover to the intervention arm of the study, and for the period from month 24 to 36, instil 0.01% atropine eye drops once nightly. An additional monitoring and re-treatment phase is planned (Phase 3), the design of which will be informed by (i) the outcomes of Phase 1, (ii) consultation with our TSC and SAC, (iii) regulatory approval and (iv) funding availability. To ensure the study population is representative of the Irish population, recruitment is capped by ethnic background in accordance with ethnic classifications in the 2016 Irish Census ^45^.

### Randomisation

The Mater Misericordiae University Hospital (MMUH) Clinical Research Centre (CRC) will carry out the stratified randomisation procedure. Participants are allocated to one of the two intervention groups according to a 2:1 treatment to control ratio to maximise recruitment success. Allocation is carried out using block randomisation and stratified according to baseline refractive error (<-3D or ≥ -3D). A randomisation list for each strata will be prepared by an independent statistician and will be stored within the CRC.

A key advantage of block randomisation is that treatment groups tend to be uniformly distributed by key outcome-related characteristics and the 2:1 assignment of participants remains similar at all times ^46^, even if the full quota of participants are not recruited into the clinical trial. Participants are randomised to a study number unrelated to treatment assignment. Once officially enrolled on the trial, participants receive a unique study identification number at the baseline visit that is pre-randomised to one of the intervention arms.

### Masking

Study treatment assignment is double masked during the first 24 months. Atropine and placebo eye drops are packaged identically before delivery of the investigational product to the trial centre, so that the investigator and participants are unable to identify the contents. Labels on the box containing the ampoules have a batch number (that does not indicate to the investigator whether it is atropine or the placebo), study reference number, participant ID, trial sponsor’s name and contact number, investigator name, site address, expiration date of the eye drop, storage instructions, and a statement informing the participant that the drop is for clinical trial use only and is not to be ingested.

No interim analyses are planned. The randomisation code is only broken after all participants have completed the 24-month visit and all data have been subjected to a “Blind Review”. This pre-analysis review, masked to treatment, covers, for example, decisions concerning the exclusion of participants or data from the analysis sets, the checking of possible transformations and definitions of outliers, the addition to the model of important covariates identified in other recent research, and other factors that might be of relevant to the data analysis. Decisions made at this time are described in a report and distinguished from those made after the study statistician has had access to the treatment codes when final decisions can be adequately taken, as masked decisions generally introduce less potential for bias. Only the DSMC has access to the randomisation list to determine allocation to atropine or placebo within the appropriate strata, as well as to facilitate interim trial safety analyses. To reduce performance and ascertainment bias after randomisation, measures are taken as far as is practical to maintain investigator masking.

### Storage and disposal of study treatment

The trial intervention drug and placebo eye drops are securely stored at the clinical trial site (CERI). Only the study investigators and the senior research assistant have access to the eye drops. Study medication, monitored constantly with Bluetooth temperature sensors, is stored at room temperature of 20°C to 25°C, with minimal excursions permitted. A room temperature log is maintained at the study site. Subjects and their parent/guardian are also instructed to store the eyedrops at room temperature with no exposure to extreme temperatures (such as refrigeration).

Parents are provided with a study treatment kit set at each visit. Each kit contains a 6-month supply of eye drops consisting of 200-unit dose ampoules. Each 6-month treatment kit consists of two 3-month boxes and each box consists of 20 aluminium foil pouches. Each foil pouch consists of 5-unit dose ampoules. Parents are instructed to open one ampoule per day and administer the eye drop into both eyes of the participant once nightly. After instillation of the eye drop, the open ampoule and the remaining contents should be placed in the provided receptacle that is returned to the study centre for accounting and proper disposal. All returned ampoules are deposited in appropriately labelled hazardous waste containers and disposed of in keeping with chemical waste management policies and procedures. Parents are provided with an information sheet on correct eyedrop use. This includes an instruction to start a new box each month irrespective of how many ampoules remain in the previous box. A contact phone number is also provided in the participant’s study information pack as well as the product label, in case the parent has any queries.

### Compliance

The study medication is provided to participants at each study visit during their treatment period. Parents sign to confirm they have received the eye drops once dispensed and are asked to bring trial boxes containing their remaining unused ampoules on all visits after the baseline visit. Parents are provided with a list of suggestions to encourage adherence, and are asked to create a nightly calendar reminder, as well as a calendar log of date and time of eye drop instillation on their phone. Regular phone and text message contact is maintained with parents throughout the study duration. Participants and their parents are questioned regarding adherence at each visit and are constantly reminded about the importance of adhering to the treatment protocol.

Participants are encouraged to attend all study visits. Follow up visits are scheduled at 6, 12, 18, 24, 30 and 36 months after the baseline randomisation visit, +/- 2 weeks. Thus, clinic visits are scheduled from up to 2 weeks before the check-up date is due. If the participant does not attend the visit, a new visit is rescheduled within 4 weeks in the first 12 months of the trial and within 6 weeks thereafter. Participants are informed that they may be removed from the trial if they fail to attend within the defined time period. If a participant is dropped from the trial for this, or any other reason, arrangements are made for collecting used and unused ampoules.

The study is to be conducted using an intention-to-treat basis. The level of compliance with eyedrop use is quantified by questionnaire (self-report), the eyedrop use calendar log and quantification of returned ampoules (extended data ^47^). There is no minimum eyedrop insertion compliance criterion that would cause removal from the trial, but compliance is controlled for in statistical analyses and used as a measure of acceptability of the treatment in our secondary objectives.

Any suspected non-compliance or improper eye drop use results in encouragement to parents and children as well as offering an electronic daily reminder system for families who have not instilled the drop as instructed. Evidence of over use is also discussed with participants and they are re-instructed on proper use and compliance with the once-nightly protocol.

### Sample size calculation

Based on available atropine trial data from Singapore ^17^, along with Pirenzepine trials data from the USA ^48^, myopia progression in the atropine treatment group is postulated to be -0.25D per annum, with a standard deviation of ±0.3D at two time points (12 months and 24 months). Power analysis of these data resulted in a projected effect difference of 50% between the intervention versus the placebo control group on myopia progression rate. The primary outcome for this study is the progression of myopia over the 2-year trial duration sampled every 6 months. The analyses of progression of myopia will be a 2x5 repeated measures mixed analysis of variance (ANOVA) with the factors of group (placebo and intervention) and time (0 months, 6 months, 12months, 18 months, 24 months). Conducting a power analysis for the repeated measures (RM) ANOVA, anticipating a conservative effect size of 0.3, an alpha of 5%, a power of 95%, with two groups, five repeated measurements and a correlation among the repeated measures of 0.9 resulted in a sample size of 136 with a critical F value of 3.91179. Allowing for a high potential attrition rate (based on the fact that no trial has been conducted to completion in a European population, in whom the acceptability and motivation for long term use remain unknown), a total of 250 children will be recruited. Of these, 167 children will be randomised to the intervention group and 83 to the placebo group.

The study is powered for the primary outcome. However, for secondary measures or subgroup analyses, the MOSAIC team will lead a planned meta-analysis of these data combined with data from ongoing trials in the UK (CHAMP-UK: ISRCTN99883695) and Australia (ATOM-Australia: ACTRN12617000598381). Such a meta-analysis will provide additional statistical power for subgroup and secondary analyses and allow a more definitive exploration of the mechanistic components of atropine’s influence on myopia progression.

### Eligibility criteria

Only participants between the ages of six to 16 years inclusive are eligible to participate in the trial. Additional inclusion criteria comprise:

1. Refractive criteria

- A spherical equivalent refractive error of -1.0D or worse
- Evidence of myopic progression over the preceding year
- Astigmatism less than -2.50D and the least myopic meridian must be more myopic or equal to -0.50D
- Corrected visual acuity of 0.2 logMAR (logarithm of the minimum angle of resolution) or better in both eyes
- Normal binocular vision, no history of amblyopia or strabismus
- No previous pharmaceutical or optical myopia control interventions

2. Health criteria

- Normal intraocular pressure (IOP) (<= 21mmHg)
- Normal ocular health, with no history of glaucoma or any other ocular diseases or ocular surgery
- No significant or severe corneal damage or scarring
- Good general health with no history of myasthenia gravis or any cardiac, respiratory, kidney or urinary disease or dysfunction
- No known allergy to atropine, cyclopentolate hydrochloride and/or proxymetacaine hydrochloride
- A negative pregnancy test for females with childbearing potential

3. Capacity criteria

- Willingness to commit to the duration of the clinical trial and to accept the possibility of randomisation to the placebo arm.
- Ability of the participant (or parent/guardian) to provide written informed consent.

### Study recruitment

Participants are recruited into this trial through a variety of channels, each of which were established prior to commencement of the pre-trial phase and are continued throughout the study until recruitment is complete. These avenues included:

1. National and local ophthalmic professional engagement: information leaflets and flyers (containing clinical trial information including trial eligibility criteria, see extended data ^47^) were sent to optometrists in the community advising them of the study and to relate this information to potential participants and their parents/guardians ^49^. Information packs (containing information leaflets for optometrists and for parent, as well as flyers, see extended data ^47^) were also distributed at various ophthalmic continuing professional development and networking events. Flyers are now displayed in clinical practices and in the National Optometry Centre, Dublin, Ireland ^49^.
2. Professional associations such as the Association of Optometrists Ireland and Irish College of Ophthalmologists were approached to seek and facilitate involvement of their members in providing information on the trial to potential participants in hospital, community practice and public health settings.
3. TU Dublin collaborations: The Access and Civic Engagement Office in TU Dublin was utilised to promote engagement with local schools, while additional school contacts were used to engage schools outside Dublin.
4. Organised advertising campaign: a parallel media campaign promoted awareness of the trial. National and local media were informed of the trial. Radio, newspaper and online advertisements were disseminated. ( http://www.ceri.ie/assets/information-leaflet_myopia_ceri.pdf, https://www.rte.ie/brainstorm/2018/0904/991568-what-tech-is-doing-to-your-eyesight/, https://www.irishtimes.com/life-and-style/health-family/lifestyle-linked-to-huge-increase-in-short-sightedness-1.3397726,)
5. A dedicated project website ( http://www.ceri.ie/myopia-control.html) and social media presence ( https://www.facebook.com/pg/ceri.ie/posts, https://twitter.com/ceri_dit?lang=en, http://hotsta.org/ceri.ie) maintained by the project senior research assistant provided information on the study and promoted the study.

### Informed consent

In compliance with the National Consent Policy of Ireland (2013 -16 Document reference QPSD-D-026-1), parental consent and child assent (dependent on child age and maturity) is required before any trial-related procedures are undertaken. Study investigators ensure that parents and children understand the trial completely including all information on the participant information leaflet. All study information is available in a child-accessible format. Participants are informed if they are eligible at the baseline visit. All participants are given the opportunity to ask any questions about the study prior to parental consent and child assent being obtained and may withdraw consent at any time. Participant consent is collected in hardcopy and a copy is provided to the participants (extended data ^47^).

### Study visits

At the baseline visit, each participants’ eligibility is confirmed, the ability of the parent to instil and participants’ ability to tolerate the instillation of an artificial tear eye drop is assessed. The participant or parent/guardian is given the allotted atropine or placebo eye drops to take home and instil one drop every night into both eyes for the trial duration. After the baseline visit, study visits are conducted at 6, 12, 18, 24, 30 and 36 months. Each visit takes approximately 90 minutes. Table 1 summarises the clinical procedures conducted at each visit.

**Table 1.** MOSAIC clinical procedures during Phase 1, 2 and 3 of the trial. Abbreviations: Tx, Treatment; *, Only for those participants who crossover to atropine treatment; **, Only for those participants undergoing re-treatment

Procedures	Phase 1					Phase 2		Phase 3	
	Visit 1 Baseline	Visit 2 6 months	Visit 3 12 months	Visit 4 18 months	Visit 5 24 months Finish tx/ crossover	Visit 6 6 months into crossover & washout	Visit 7 12 months into crossover & washout	Visit 8 6 months crossover washout, monitoring/ re-tx	Visit 9 12 months crossover washout, monitoring/ re-tx
Inclusion/Exclusion Criteria	✓	✓	✓	✓	✓				
Informed Consent	✓								
Medical History	✓								
Review of Medical History		✓	✓	✓	✓	✓	✓	✓	✓
Heart Rate	✓	✓	✓	✓	✓	✓ *	✓ *		
Height	✓	✓	✓	✓	✓	✓	✓	✓	✓
Weight	✓								
Pregnancy Test	✓								
Parental Autorefractor	✓								
Visual Acuity	✓	✓	✓	✓	✓	✓	✓	✓	✓
Accommodative Lag/ Facility/Amplitude	✓				✓		✓		✓
Near Point of Convergence	✓				✓		✓		✓
Near VA	✓				✓		✓		✓
IR Pupillometry	✓	✓	✓	✓	✓	✓	✓	✓	✓
Tonometry	✓	✓	✓	✓	✓	✓	✓	✓	✓
Objective Refraction	✓	✓	✓	✓	✓	✓	✓	✓	✓
Off-Axis Refraction	✓	✓	✓	✓	✓	✓	✓	✓	✓
Fundus Image	✓	✓	✓	✓	✓	✓	✓	✓	✓
Corneal Topography	✓	✓	✓	✓	✓	✓	✓	✓	✓
Slit lamp Assessment	✓	✓	✓	✓	✓	✓	✓	✓	✓
Ocular Biometry	✓	✓	✓	✓	✓	✓	✓	✓	✓
Optical Coherence Tomography	✓	✓	✓	✓	✓	✓	✓	✓	✓
Confocal Scanning Laser Ophthalmoscopy	✓	✓	✓	✓	✓	✓	✓	✓	✓
Adverse event monitoring		✓	✓	✓	✓	✓	✓	✓	✓
Compliance check		✓	✓	✓	✓	✓ *	✓ *		
Questionnaires		✓	✓	✓	✓	✓			
Dispensing of atropine or placebo	✓	✓	✓	✓	✓	✓	✓ **	✓ **	

### Description of study assessments

***Demographics and inclusion/exclusion criteria*.** At the initial screening visit all parents are required to complete a demographic and health questionnaire on behalf of their child (extended data ^47^). Demographic information includes age, sex and race. Iris colour is documented ^50^. Medical and ocular history, including the participant’s GP and optometrist name and address, is also collected. To participate in the trial, participants must satisfy all the inclusion and exclusion criteria outlined.

***Medical and surgical history*.** A detailed medical and surgical case history is undertaken in order to ensure that all trial participants are in good general and ocular health and comply with the study inclusion and exclusion criteria, including any history of ocular surgery, amblyopia and patching as well as detailing any allergies to atropine sulphate, hydroxypropyl methylcellulose, proxymetacaine hydrochloride or cyclopentolate hydrochloride. 

Heart Rate is measured at baseline and every 6 months thereafter until the final study visit. Heart rate is measured with a heart rate monitor after approximately 3 minutes of rest in the seated position. The participant is excluded if heart rate is persistently (for more than 10 minutes) > 120 beats per minute at any study visit.

Females of childbearing potential are required to provide confirmation of last menstrual period and a negative pregnancy test. Sexually active participants are advised to use an acceptable, effective form of contraception until cessation of treatment.

### Pre-cycloplegia measures

***Visual acuity*.** Participants’ corrected visual acuity is measured using a randomised letterset of the MultiQuity (MiQ 720) computerised logMAR chart.

***Accommodation and convergence*.** Amplitude of accommodation (AoA) and near point of convergence (NPC) are measured non-invasively with a RAF (Royal Air Force) rule using the subjective push-up technique. Measurements are taken both monocularly and binocularly for AoA and binocularly for NPC. Lag of accommodation to a 40cm (2.5D) target is measured using the Grand Seiko WAM 5500 open field autorefractor. Accommodative facility is measured using ±2.00D flippers at near.

***Near VA*.** Near visual acuity is documented to monitor any changes in visual performance. Near visual acuity is determined as the smallest character size that can be read at 25cms.

***Infrared pupillometry*.** The Topcon ALADDIN optical biometer is used to measure scotopic and photopic pupil size and pupil reactivity for each participant at each visit. Atropine causes pupillary dilation, particularly at higher concentrations used in previous trials. This measure is taken pre- and post-cycloplegia and is used to determine the physiological impact of 0.01% atropine on pupillary function as well as to confirm cycloplegia.

***Tonometry*.** An Icare tonometer is used to calculate the intraocular pressure in both eyes at each study visit pre- and post-cycloplegia. This measure is taken at all visits as a safety precaution for participants.

### Post cycloplegia measures

***Objective refraction*.** Refractive error and peripheral refractive error are measured by cycloplegic auto-refraction at the baseline visit using the Grand Seiko WAM 5500 open field autorefractor. Change in spherical equivalent refraction and peripheral refraction (30° nasal and temporal) are measured by cycloplegic auto-refraction at all subsequent visits. One drop of 1% cyclopentolate is instilled 30 minutes prior to auto-refraction. Cycloplegia is confirmed using measures of pupil size, pupil dynamics and near visual acuity. If both pupils are not dilating and amplitude of accommodation is not reducing after 15 minutes, a second drop of 1% cyclopentolate is instilled.

***Choroidal thickness*.** Swept source optical coherence tomography (OCT) provides a significant improvement over conventional OCT, in particular, there is better penetration of the deeper layers of the eye including the choroid. The 7×7 macula scan on the Topcon DRI OCT Triton, facilitates automated choroidal thickness mapping at each study visit.

***Retinal vasculature*.** The 7×7 macula and angiography scans on the Topcon DRI OCT Triton allows imaging of the central and peripheral retina for detailed analysis of change in retinal vessels in relation to ocular growth and also to examine and monitor retinal health throughout and after the clinical trial.

***Slit lamp assessment*.** The slit lamp is used to perform a complete examination of the external eye as a safety precaution throughout the trial in case of sign of allergy or external abnormality. This includes examination of the cornea, conjunctiva, iris, pupil, lids and adnexa, relative to the Brien Holden Vision Institute grading scale where appropiate ^51^. Results are recorded at each visit study visit, including visits during the washout period. As the study medication contains phosphates, inclusion in the study requires a healthy cornea with no evidence of corneal damage or scarring. Cases of corneal calcification have been reported very rarely in association with the use of phosphate-containing eye drops in some patients with significantly damaged corneas ^52, 53^.

***Ocular biometry*.** The Topcon ALADDIN optical biometer is used to determine the axial length, anterior chamber depth, pupil size, keratometry, dynamic pupil measurements, white to white and lens thickness at the baseline visit. Changes in dynamic pupil measurements, ocular axial length, anterior chamber depth and/or lens thickness are documented by repeat measurements at all subsequent visits.

***Adverse event assessments*.** A detailed adverse events assessment will be conducted at each visit, which includes specific evaluation as to the experience of known adverse side effects of topical atropine which can include:

- Reading difficulties: some degree of cycloplegia is anticipated while using 0.01% atropine.
- Glare: an increase in pupil size and loss of reactivity is expected while using 0.01% atropine.
- Other, less common and rare ophthalmic adverse effects of atropine may include conjunctival irritation, follicular conjunctivitis, increased intraocular pressure (especially in patients with closed-angle glaucoma), and swelling of the eyelids. Participants and parents are provided with information describing the clinical signs of the above adverse effects, and given appropriate instruction as to appropriate management in each case.
- Systemic adverse effects: Tachycardia, dryness of the mouth, flushing, anhidrosis, heat intolerance or impaired temperature regulation, hypersensitivity-associated skin rashes. Participants and parents are provided with information describing the clinical signs of the above adverse effects and given instruction as to appropriate management in each case. Heart Rate is measured at baseline and every six months thereafter until month 24. Heart rate is monitored at month 30 and 36 for those participants who have crossed over to atropine treatment.

The detailed examination carried out at each study visit is used to determine any symptomatic or asymptomatic adverse responses to atropine use. A detailed case history is also used to determine the presence of adverse events attributable to the therapeutic intervention. Occurrence of adverse events are recorded on study-specific adverse report forms. The DSMC review all adverse events and safety data on a quarterly basis, or more frequently if required by any emerging safety concerns during the study.

In the unlikely scenario of a serious adverse event, our team includes a paediatric ophthalmic surgeon, who will make arrangements for ophthalmic service provision with ophthalmology and medical colleagues, as appropriate, to manage ophthalmic adverse events/effects as may be required. Non-ophthalmic adverse events are managed through appropriate public health channels including the family physician, emergency services, accident and emergency unit or other hospital services as may be required. In the event an unexpected and serious adverse side-effect is confirmed to be directly attributable to the investigational product, the DSMC, TSC and Study Sponsor will liaise with the research ethics committee (REC) and Health products Regulatory Authority (HPRA) to determine the appropriate course of action.

### Questionnaires

***Lifestyle questionnaire*.** At each study visit parents are asked to complete a lifestyle questionnaire to quantify lifestyle habits including outdoors exposure, sports participation, near work including use of digital technology (see extended data ^47^) ^49^.

***Quality of Life Impact questionnaire*.** The impact or burden of atropine treatment on the child and the family is assessed using a validated and atropine specific quality of life questionnaire, adapted from the Parent and New Child Amblyopia Treatment Index (ATI) questionnaires used with higher atropine concentrations ^49, 54^.

### Statistical analysis

***Primary efficacy endpoint*.** The primary outcome, in line with previous studies ^17, 55^, will be myopia progression amongst study groups, primarily the difference in change in myopic refractive error (in dioptres) from cycloplegic autorefraction from baseline to month 24. The analysis will rely on parametric analysis of variance (ANOVA), to control for the effects of the other continuous variables that are not of primary interest. The treatment difference in response rate and its 95% confidence interval (CI) will be provided.

***Secondary efficacy endpoints*.** Univariate analysis will be performed to evaluate the group effect on mean change in axial length over the 2-year period. Multivariate regression analyses will be used to assess the association between change in axial length and progression of myopia while adjusting for treatment effects. The difference in myopic refractive error from cycloplegic autorefraction and axial length between the treatment groups from baseline to visit 6 will be analysed using RM ANOVA.

The percentage of participants in each treatment group that progress <0.25D, 0.25D≤0.75D and >0.75D from baseline to visit 6 will be displayed in cross tabulations.

A post hoc analysis, with Bonferroni correction to account for any type 1 errors, will be carried out to determine at what time-point over the 24-month treatment period atropine was most effective.

The distribution of scores from the quality of life questionnaire is calculated at each visit for all treatment groups. At the 24-month visit the scores for each visit will be calculated and analysis of variance (ANOVA) and suitable post hoc testing will be used to determine any statistically significant differences between the responses at each visit as well as between groups. Safety and tolerability data will be presented in tables of descriptive statistics and frequency distribution. RM ANOVA will be used to detect changes in anterior chamber depth, crystalline lens thickness, retinal nerve fibre layer thickness and choroidal thickness between treatment groups. A statistical significance level of p < 0.05 is adopted throughout the analysis.

Adjustment for baseline characteristics will be performed by analysis of covariance (ANCOVA). Several comparisons will ensue based on further stratification by a) sex; b) age; c) ethnicity; d) baseline refractive error and e) baseline axial length, using data pooled from both eyes, assuming the sample size is sufficient. The primary statistical analysis will be based on the intention-to-treat principle, but supported by additional per-protocol analyses.

### Trial steering and oversight

Three committees have been established to provide independent oversight and steer the strategic direction of the trial.

### Data Safety Monitoring Committee

A three-member independent DSMC has been established to oversee the safety of trial participants. The establishment of the DSMC is justified given the paediatric population under investigation and the need to detect any potential harm to participants as early as possible, even though the anticipated potential for harm in this trial is very low. Safety monitoring will be the major task for the appointed DSMC. All members of the DSMC are completely independent of the trial and have no possible financial or other potential conflict of interest in the study. The DSMC is composed of a statistician with expertise in biomedical research, a paediatric ophthalmologist and an ophthalmologist with medical and surgical retina expertise (chairperson).

### Trial Steering and Scientific Advisory Committees

In addition to the DSMC, a TSC and SAC has been appointed to contribute to the design and conduct of the trial. The TSC includes individuals with expertise and involvement in other atropine trials for myopia control. The SAC includes international scientists with expertise in the exploration of efficacy, safety and mechanistic aspects of myopia and its control. The trial investigators are represented on the Steering Committee by JL and IF. Together the co-PIs maintain responsibility for all study design, implementation and oversight across all facets of the study. The TSC hold teleconference meetings as required. Meetings address trial strategy, trial protocol and matters arising from the various work packages including staff and other resource concerns, data collection and all operational issues as they arise. The scientific and clinical members of the steering committee have the responsibility of informing the clinical protocol required to effectively and safely implement the trial, including determination of patient eligibility, examination protocol, data capture and recording protocol, inter-professional communication protocol, and decision review protocol. Members of the TSC and SAC are identified in Figure 1.

### Regulatory approval

Regulatory approval for MOSAIC was granted by the Health Products Regulatory Authority (HPRA), reference CT0900/622/001. Clinical trials in Ireland are currently governed by the European Communities (Clinical Trials on Medicinal Products for Human Use) Regulations, 2004, SI No 190 of 2004, transposed into Irish law the provisions of Council Directive 2001/20/EC. 

### Ethics

Ethical approval for the study was granted by the Research Ethics Committee (REC) at the MMUH, Dublin, Ireland (reference 1/478/81), and by the REC at TU Dublin, Ireland (reference 16-45). MOSAIC adheres to the tenets of the Declaration of Helsinki and follows the full code of ethics with respect to participant recruitment, participant testing and General Data Protection Regulations (GDPR, effective May 2018).

As required under the EU Directive on Clinical Trials (European Union - Directive 2001/20/EC), parental or guardian consent is obtained as required for clinical trials involving minors, who are defined in this case as persons under the age of 16 years. MOSAIC is carried out within the framework of Children First: National Guidance for the Protection and Welfare of Children (DCYA, 2011). Child assent is also obtained in recognition of the importance of the voice of the child in this type of research ^56^.

### Dissemination of findings

Findings and underlying data from MOSAIC will be disseminated at appropriate conferences, through scientific publications and published in open access format on the TU Dublin ARROW repository. Findings will also be shared online through social media channels, blogs, and publications, as well as through print, broadcast and at stakeholder events.

### Study status

Recruiting will being June 2019 and continue through to March 2020.

## Discussion

The study that has principally helped to define the approach and methodology of MOSAIC is the ATOM2 study conducted in Singapore ^18, 57^. The ATOM2 study was designed to examine the dose:response characteristics of atropine in terms of its positive impact on myopia progression, and its negative impact in terms of blurred near vision and photophobia, and demonstrated a beneficial effect of 0.01% atropine on myopia progression without the adverse effects noted at higher concentrations ^18^.

Specific design limitations of ATOM2 are addressed in the MOSAIC clinical trial. These include a true placebo group, increased participant numbers in the 0.01% treatment cohort and removing the potentially confounding effects of varifocal glasses that were provided to some of the high-dose groups in the ATOM2 study.

MOSAIC will investigate the efficacy, safety and acceptability of 0.01% unpreserved atropine solution for the control of myopia in European (predominantly White) children. The recruitment strategy is a particular strength of the MOSAIC trial, in that the recruitment by ethnic origin will be capped according to the demographics within the Irish population according to the most recent census in order to ensure that results are truly representative of the Irish population. At the 2016 Irish census, 91.7% of the population identified as White ^45^. This approach will result in the recruitment of an equally high proportion of White participants with progressive myopia, a group that has yet to be adequately targeted in relation to the safety and efficacy of atropine. Future meta-analysis will also permit evaluation of unpreserved 0.01% atropine efficacy and safety compared to preserved (CHAMP-UK and WA-ATOM) 0.01% atropine. 

MOSAIC is also designed to ensure that, at least at the time of enrolment, each participant exhibits progressive myopia. The principal benefits of this feature include that (i) this allows a broader age range of eligible participants which is important in a European context where myopia onset is typically later and slower relative to in Asia, (ii) it reduces the possibility that the outcomes are confounded by variability in the proportion of naturally non-progressive myopes across treatment arms, and (iii) it thereby ensures that any differences at the completion of the trial can be attributed to atropine treatment ^58^.

MOSAIC will provide novel insights into the mechanisms affecting myopia development, as well as the mechanism of action of atropine, which still remains unclear ^59^. The MOSAIC protocol has been made available to other research groups and is closely replicated in studies in the UK and Australia, which when pooled for meta-analysis will provide increased scope to investigate the mechanism of action for atropine’s anti-myopia effect.

## Conclusion

Myopia has become a significant global public health problem. The safety and efficacy of atropine eye drops in reducing myopia progression has been demonstrated in several clinical trials in Asia; however, due to potential inter-ethnic differences, the generalisability of such results are limited. The MOSAIC trial can address significant evidence gaps that exist in relation to atropine as a myopia control intervention in a predominantly White population. MOSAIC’s extensive study design and large sample size are major advantages in determining atropine’s efficacy, mechanism of action and safety in a Western context. In addition, the planned pooling of data with similar trials in future meta-analysis will provide sufficient power for subgroup analysis by age, race, initial refraction and atropine formulation.

## Data availability

### Underlying data

No data is associated with this article.

### Extended data

*ARROW @ TU Dublin*: Myopia Outcome Study of Atropine in Children (MOSAIC): Design and Methodology. https://doi.org/10.21427/xyq9-ck53 ^47^

- 1 Parent consent and information leaflet (2).pdf (MOSAIC Parent Information Leaflet and Consent Form)
- 2. Child assent and information leaflet .pdf (MOSAIC Child Information Leaflet and Assent Form)
- 7. Eyedrop Questionnaire for Parents- BASELINE (1).pdf (MOSAIC Baseline Visit Eye Drop Questionnaire for Parents)
- Eyedrop QOL Questionnaire for Parents Month 6, 12, 18, 24 (2).pdf (MOSAIC Follow-up Visit Eye Drop Questionnaire for Parents)
- 8. Self-reported discomfort Child Sep18 (2).pdf (MOSAIC Self-Reported Discomfort Questionnaire for Child)
- 9. MOSAIC Activity diaries .pdf (MOSAIC Activity Diary)
- 5.mosaic recruitment flyer final.png (MOSAIC Recruitment Flyer)
- 10. STUDY INFORMATION PACK (1) (1).pdf (MOSAIC Study Information Pack)
- information-leaflet_myopia_ceri.pdf (Myopia Control Information Leaflet for Parents)
- information-pack_myopia_ceri.pdf (Myopia Control Information Pack for Optometrists)

### Reporting guidelines

ARROW@TU Dublin: CONSORT checklist for ‘Myopia Outcome Study of Atropine in Children (MOSAIC): an investigator-led, double-masked, placebo-controlled, randomised clinical trial protocol’. https://doi.org/10.21427/3neg-gr77 ^35^

ARROW@TU Dublin: SPIRIT checklist for ‘Myopia Outcome Study of Atropine in Children (MOSAIC): an investigator-led, double-masked, placebo-controlled, randomised clinical trial protocol’. https://doi.org/10.21427/2dbf-t103 ^36^
