# Supplementary material for: Myopia Outcome Study of Atropine in Children (MOSAIC): an investigator-led, double-masked, placebo-controlled, randomised clinical trial protocol
Source: HRB Open Res. 2019 Jul 23;2:15. [Version 1] doi: 10.12688/hrbopenres.12914.1 (PMC6973533; doi:10.12688/hrbopenres.12914.1)
Supplement: Peer review history

# Peer review: Reviewer response for version 1

**DOI:** 10.21956/hrbopenres.13992.r26704

**Published:** 2019-09-10

**Authors:** Jan Roelof Polling

**Affiliations:** 1 Department of Ophthalmology, Erasmus University Medical Centre, Rotterdam, The Netherlands; 2 Department of Optometry and Orthoptics, University of Applied Science, Utrecht, The Netherlands

The paper describes a trial for myopia control with low dose atropine in Europeans. There is a need for these trials as the efficacy of low dose atropine is disputed, not only in Western but also in Asian children. I have a few questions for the authors for clarification of the introduction, methods and current results.

 The introduction states that the ATOM2 achieved excellent control over refractive error progression with atropine 0.01%. We could debate what would be excellent control however the ATOM2 study found for this group completely no control over AL progression. Actually, there was more progression in the treatment group than in the control group. The authors should discuss the dispute rather than the effects in the non-RCT setup of ATOM after the 2 RCT years. One should carefully study the facts rather than to praise the partial effects in RCT’s or cohort studies. It is also a fact that the LAMP study only showed 12% AL growth reduction. Nevertheless this new study should highlight both SER and AL and discuss openly that in myopia management the AL reduction is the measure we are striving for.

 The age range from 6-16 is wide and it is known that myopia progression and especially AL progression diminishes with age. It is unfortunate that the trial committee did not stratify for age when randomizing. Could the authors discuss the problems that could arise with the outcome regarding to age and treatment effect vs. normal progression?

 Figure 2 shows a flowchart of the patients in the study. This paper would be the place to discuss what happened to the excluded patients. For future myopia management studies it is very relevant information. Could the authors explain in detail all reasons for not participating in this study? Phase 3 describes a 0.02% doses however this is not discussed earlier, please comment.

 The eligibility criteria lists evidence of myopic progression over the preceding year. How is this quantified? Glasses, compared to subjective measurement or is cycloplegia required? AL? Also listed is normal binocular vision. It is not listed with what test normal correspondence is tested. Is ocular motility or cover testing preformed? TNO test?

 Cycloplegia is confirmed with pupil size, what should be the pupil size and what should be the amplitude of accommodation reduction?

I confirm that I have read this submission and believe that I have an appropriate level of expertise to confirm that it is of an acceptable scientific standard, however I have significant reservations, as outlined above.

# Peer review: Reviewer response for version 1

**DOI:** 10.21956/hrbopenres.13992.r26705

**Published:** 2019-08-27

**Authors:** Jason C. Yam

**Affiliations:** 1 Department of Ophthalmology and Visual Sciences, Chinese University of Hong Kong, Hong Kong, China

I read with great pleasure on the “Myopia Outcome Study of Atropine in Children (MOSAIC): an investigator-led, double-masked, placebo-controlled, randomized clinical trial protocol.” The authors are to be congratulated on launching this important trial. 

 Summary of the MOSAIC trial.

1. A double-masked, placebo-controlled, randomized clinical trial, on 250 Caucasian children aged 6 - 16.
2. Randomized into two groups: 1) atropine 0.01% once nightly; 2) placebo group once nightly; in 2:1 ratio, for 24 months during phase 1.
3. Atropine treatment group will be washed out, and placebo group crossovered to 0.01% atropine group during 24 to 36 months during phase 2.
4. Followed up at every 6-months interval under a standardized protocol.
5. Detailed documentation of parameters, including cycloplegic SE, AL, pupil size, accommodation by RAF, near and distance VA, corneal topography, Choroidal thickness by OCT, fundus images, questionnaires for side effects etc.
6. Further re-treatment protocol will be planned depending on the outcome of initial phases.

This is a well-designed randomized control trial to answer important clinical questions in using low-concentration atropine for myopia control. First, this serves as the first RCT of using low-concentration atropine in European populations. Second, the study has a two-year placebo period, which will provide more robust evaluation on atropine 0.01%. Third, detailed investigations including biometric measurement, OCT, retinal image will provide valuable information on the potential anti-myopia mechanism of low-concentration atropine.

 I have a few comments for the authors’ consideration for further enhancement.

 Eligibility criteria:

 No previous “or current” pharmaceutical or optical myopia control interventions. Those who have combined treatment should be excluded.

 Randomization:

 The current randomization is stratified according to baseline refractive error. Given the wide range of subjects in the study, 6-16, I would suggest to consider to have additional stratification by sex and age.

 Near VA:

 It may be better to specify that “Near visual acuity was assessed using best-corrected distance spectacle correction”.

 Post cycloplegia measurement:

 It is important to specify in detail the cycloplegia protocol, as this will influence the clinical outcomes significantly.

 Adverse event assessments:

 Need to document the number of subjects wearing photochromic lens.

 Questionnaires:

 In my opinion, the authors may consider adding a set of visual function questionnaires which may evaluate better how the visual function under atropine treatment is being affected in the daily life. This is optional.

 Phase two protocol.

 I would suggest for authors’ consideration for enhancement. Again, this is optional at the discretion of the authors.

 For phase two, the authors may consider to re-randomize atropine 0.01% group into continued 0.01% atropine, and washout, at 1:1 ratio, and placebo group remained crossovered to 0.01% atropine group. This will end up having three groups at 1:1:1 ratio of:

1. Atropine 0.01% continued for three years.
2. Atropine 0.01% for two years, followed by washout one year.
3. Placebo group for two years, followed by atropine 0.01% for one year.

This may better evaluate the rebound effect of 0.01% atropine.

I confirm that I have read this submission and believe that I have an appropriate level of expertise to confirm that it is of an acceptable scientific standard.

# Peer review: Reviewer response for version 1

**DOI:** 10.21956/hrbopenres.13992.r26706

**Published:** 2019-08-12

**Authors:** Sara McCullough

**Affiliations:** 1 Optometry and Vision Science Research Group, School of Biomedical Sciences, Biomedical Sciences Research Institute, University of Ulster, Coleraine, UK

This article describes the methodology for recruitment and data collection for the Myopia Outcome Study of Atropine in Children (MOSAIC), a double-masked, placebo-controlled, randomised clinical trial. It aims to determine the efficacy, safety and mechanism of action of 0.01% atropine to slow the progression of myopia in a predominantly white, European population of children aged six to 16 years of age. Myopic children with progressing myopia will be recruited to the study and will be randomly assigned to either the treatment group (0.01% atropine) or the placebo group and will be reviewed every six months for a trial period of 2 years. The following year will either be a washout period for the treatment group or the placebo group will receive 0.01% atropine daily. There is clear rationale for the study with the worrying increase in prevalence of myopia across Europe and the need for a better understanding of treatment therapies for controlling myopia progression. The majority of previous clinical trials of atropine of varying concentrations have been conducted in Asian populations where the progression of myopia differs to white, European children, therefore similar studies need to be replicated to determine specific population efficacy which will be answered by the proposed study design. The study methods are clearly described although I had a few specific queries which could be addressed/clarified by the research group to allow replication by others. There were also some discrepancies between the ‘extended data’ provided that didn’t tie up with that published in the article. 

 These include:

1. You mention in your flaws of the ATOM2 study the confounding effects of varifocals prescribed to some of the high-dose participants within their study but you do not describe what refractive correction the participants within your own study will wear. Will this be full refractive correction with single vision spectacle lenses/contact lenses/other? No details are described of whether spectacles will be updated to the new correction where a difference is found within the trial; this may be important with regard to potential under-correction of myopia. 
2. Within your eligibility criteria, it is not clear how much myopic progression over the preceding year is considered enough? Within the information pack for parents the inclusion criteria states that progression of -0.50D in the preceding year is required but this is not mentioned in the publication. It would be helpful to know how much progression was occurring prior to participation in the study and consider this in analyses. There are no references to why the eligibility criteria were chosen; are these replicated from another RCT? It is perhaps unusual to see the inclusion criteria extend to 16 years of age as the rate of progression of myopia will be fairly slow at this age and the age range doesn’t align with that of other studies (e.g. ATOM 6-12 years). In the parent information pack the eligibility criteria states that participants need to have myopia of <-1.50D but in the article under review it is -1.00D or worse; why the discrepancy? It is also mentioned within the parent information leaflet that inclusion requires a difference between the two eyes of less than 1D but this isn’t mentioned in the article, was this considered as an inclusion/exclusion criteria or not?
3. You mention that good general health was a requirement for inclusion, did you consider syndromes, e.g. Down syndrome, Marfan’s, Stickler’s that may have high levels of myopia and respond differently to treatment? If so, were these exclusion criteria? Were diabetics excluded from participation due to myopia shifts that can occur with poor control?
4. I also found a few discrepancies in the child assent and information leaflet. You state that “the drops, which don’t sting or blur your vision, will need to be put into each eye every evening” but yet you are asking the child to comment within a self-report discomfort questionnaire about these symptoms. It also states that cyclopentolate 0.5% will be used to measure the participant’s need for glasses but in the article you suggest you are using cyclopentolate 1.0%.

I confirm that I have read this submission and believe that I have an appropriate level of expertise to confirm that it is of an acceptable scientific standard.
